# Breast Cancer Medications and Vision: Effects of Treatments for Early-stage Disease

**DOI:** 10.3109/02713683.2011.594202

**Published:** 2011-08-05

**Authors:** Alvin Eisner, Shiuh-Wen Luoh

**Affiliations:** 1 Women's Health Research Unit, Department of Obstetrics & Gynecology, Oregon Health & Science University, Portland, Oregon, USA; 2 Division of Hematology and Medical Oncology, Knight Cancer Institute, Oregon Health & Science University, Portland, Oregon, USA; 3 Portland VA Medical Center, Portland, Oregon, USA

**Keywords:** Aromatase inhibitor, Dry eye, Estrogen, Retina, Short-wavelength-sensitive cones, Tamoxifen

## Abstract

This review concerns the effects on vision and the eye of medications prescribed at three phases of treatment for women with early-stage breast cancer (BC): (1) adjuvant cytotoxic chemotherapy, (2) adjuvant endocrine therapy, and (3) symptomatic relief. The most common side effects of cytotoxic chemotherapy are epiphora and ocular surface irritation, which can be caused by any of several different regimens. Most notably, the taxane docetaxel can lead to epiphora by inducing canalicular stenosis. The selective-estrogen-receptor-modulator (SERM) tamoxifen, long the gold-standard adjuvant-endocrine-therapy for women with hormone-receptor-positive BC, increases the risk of posterior subcapsular cataract. Tamoxifen also affects the optic nerve head more often than previously thought, apparently by causing subclinical swelling within the first 2 years of use for women older than ∼50 years. Tamoxifen retinopathy is rare, but it can cause foveal cystoid spaces that are revealed with spectral-domain optical coherence tomography (OCT) and that may increase the risk for macular holes. Tamoxifen often alters the perceived color of flashed lights detected via short-wavelength-sensitive (SWS) cone response isolated psychophysically; these altered perceptions may reflect a neural-response sluggishness that becomes evident at ∼2 years of use. The aromatase inhibitor (AI) anastrozole affects perception similarly, but in an age-dependent manner suggesting that the change of estrogen activity towards lower levels is more important than the low estrogen activity itself. Based on analysis of OCT retinal thickness data, it is likely that anastrozole increases the tractional force between the vitreous and retina. Consequently, AI users, myopic AI users particularly, might be at increased risk for traction-related vision loss. Because bisphosphonates are sometimes prescribed to redress AI-induced bone loss, clinicians should be aware of their potential to cause scleritis and uveitis occasionally. We conclude by suggesting some avenues for future research into the visual and ocular effects of AIs, particularly as relates to assessment of cognitive function.

## INTRODUCTION

This review will discuss the effects on vision and the eye of medications commonly prescribed for women diagnosed with early-stage breast cancer (BC). After this introduction, the text will be divided into three main sections, each corresponding to a different treatment phase: (1) adjuvant chemotherapy administered for months shortly after a BC diagnosis and surgery, (2) adjuvant endocrine therapy used thereafter for years in order to reduce the risk of BC recurrence, and (3) supplemental medication used as needed to alleviate or counteract the side effects of the BC medications. A final concluding section suggests directions for future research. This review will focus on medications that appear to affect the visual system in more than rare and isolated cases and are part of the standard-of-care armamentarium for early-stage BC. The term “adjuvant” denotes treatments used for reducing the risk of disease recurrence.

Because cancer medications interfere with cell growth or proliferation, they tend to be especially toxic to normal tissues: (a) that have a high rate of cellular turnover, (b) that have a high biochemical and/or anatomical vulnerability to a given medication, and (c) that are sufficiently exposed to the medication. This combination of factors probably helps explain why excessive tearing (i.e., epiphora, which involves the interplay between tear production, the ocular surface, and tear drainage)^1^–^3^ occurs as a side effect of several BC chemotherapeutic medications.^4^ Although all side effects are manifestations of chemical toxicities, some side effects - most notably those resulting from the virtual abolition of estrogen synthesis caused by inhibition of the enzyme aromatase^5^, ^6^ —might be viewed also as consequences of accelerated aging.^7^ This view is important because patients’ symptoms or complaints (e.g., increased floaters) might too often be misattributed to natural aging rather than to treatment effects.

As detailed later, two distinct classes of adjuvant endocrine medications are now widely prescribed expressly for women whose BC was identified as hormone-receptor-positive.^8^ Selective estrogen receptor modulators (SERMs) act against BC by occupying estrogen receptors (ERs), while aromatase inhibitors (AIs) act against BC by interfering with estrogen synthesis.^9^ We will feature the endocrine-therapy portion of this review for a variety of reasons, the most central being that millions of healthy BC survivors in the foreseeable future are projected to use such medications on an adjuvant basis for years at a time.^8^, ^10^–^13^ Four additional sets of reasons are listed next; each reflects the rapid evolution of basic science and clinical care.

First, overwhelming evidence is accumulating to show that estrogenic activity directly impacts a vast array of physiologic functions beyond those involving reproductive and sexual function.^6^, ^14^ Estrogen receptors (ERs) are present throughout the body, including the anterior^15^–^18^ and posterior^19^, ^20^ portions of the human eye and also in the lacrimal^18^, ^21^ and meibomian glands^18^,^22^, ^23^ responsible for protecting the surface of the eye. In fact, ERs are present throughout the central nervous system (CNS),^24^–^27^ so changes in estrogenic activity have the potential to affect central visual processing, in addition to ocular visual processing. Moreover, because estrogens are synthesized locally throughout the body, much estrogenic activity is autocrine or paracrine, rather than endocrine.^28^ These autocrine and paracrine estrogenic actions take on increased importance for post-menopausal women, whose estrogen supplies have decreased markedly^29^

Second, the longstanding standard-of-care for hormone-receptor-positive early-stage BC is changing. That is, use of the SERM tamoxifen (Nolvadex®) as adjuvant endocrine therapy is being supplanted by the use of AIs such as anastrozole (Arimidex®) or letrozole (Femara®) for women who are post-menopausal.^30^ Because the traditional 5-year period of adjuvant endocrine therapy was based on studies involving only tamoxifen,^31^ this period may be lengthened in the future, depending on the outcomes of ongoing clinical trials with AIs.^31^, ^32^ These changes in adjuvant endocrine therapy raise concerns for long-term eye health, since a prolonged period of sustained estrogen deprivation has the potential to increase the risk or severity of several age-related eye diseases or conditions, including glaucoma^33^–^37^ and macular degeneration.^38^–^41^ Moreover, because AIs are notorious for reducing bone density^42^, ^43^ any visual dysfunction- no matter how subtle- that raises the risk of falling will consequently raise the risk of fracture, even in the short-term.^44^ This cascade of events may lead to potentially devastating outcomes among at-risk BC survivors.

Third, because adjuvant endocrine medications are self-administered orally on a daily basis, they often are considered by patients to be more elective than the preceding chemotherapy^45^ which is administered intravenously in a clinical setting. Thus, adherence to adjuvant endocrine therapy often ends prematurely or becomes sub-optimal for any of a variety of reasons,^46^, ^47^ including the patient's perceived quality-of-life / side-effect tradeoffs.^48^, ^49^ The net consequence is that real-world BC recurrence rates exceed those reported in clinical trials.^50^ This set of events is especially unfortunate since greater side-effect severity signifies a better chance of BC non-recurrence,^5^, ^51^ probably because the severity of AI-induced side effects corresponds to the degree of biologically relevant estrogen suppression.^51^ Thus, the more reliably side effects can be recognized by clinicians and hence addressed, the better patients’ cancer outcomes are likely to be.

And fourth, the recent wide use of AIs provides a novel means for evaluating the ways by which estrogen may support healthy eyes and normal vision. Many women diagnosed with early-stage BC or with precancerous conditions such as ductal carcinoma in situ (DCIS) or lobular carcinoma in situ (LCIS) will use AIs but never receive cytotoxic chemotherapy,^52^, ^53^ so these women comprise a minimally confounded cohort for short- and long-term studies of estrogen deprivation in adults. Parallel studies can be conducted using animal models.

## CHEMOTHERAPEUTIC AGENTS USED SHORTLY AFTER BC DIAGNOSIS

Although it would be desirable to specify the frequencies with which ocular side effects such as “dry eye” (also called keratoconjuncrivitis sicca) result from any individual medication, several factors combine to make such estimates uncertain. First, cytotoxic chemotherapeutic agents rarely are administered entirely independently of one another. Second, toxicities typically are cumulative-dose-dependent, and side effects need not manifest immediately following the most recent treatment. Third, the physiologic responses to treatment can differ for individual patients. In fact, the recognition and prospective identification of salient individual differences provides the basis for “personalized medicine", which is being implemented in the clinic at an accelerated pace owing for a variety of scientific and technological advances.^54^, ^55^ And fourth, the frequency with which symptoms are reported depends greatly on the expectations and communications among providers, investigators, and patients.^56^, ^57^ No studies appear to have been conducted wherein validated survey tools (e.g., the Ocular Surface Disease Index (OSDI)^58^) have been used for assessing ocular-surface discomfort stemming from chemotherapy, for example. Neither do there appear to be any epidemiologic studies addressing the relation of chemotherapy to dry eye.

Bear in mind that the term *dry eye* is somewhat of a misnomer in the sense that the eyes of people with dry eye can be quite watery, typically because tears are produced reflexively to counteract the ocular surface discomfort.^59^ However, occlusion of the tear drainage apparatus may contribute occasionally to ocular irritation,^1^, ^60^ and it can allow toxic agents to remain in contact longer with the ocular surface. Dry eye syndrome is diagnosed largely according to the presence of subjective symptoms of discomfort, such as a “gritty” sensation,^61^ and it occurs most often among post-menopausal women,^3^,^62^ who coincidently are the people most likely to develop BC.^63^

As discussed next, epiphora and ocular surface discomfort may result from several different cytotoxic chemotherapy regimens, and as discussed later, there is reason to hypothesize that AI usage may contribute to dry eye. In addition to being locally irritating or otherwise bothersome,^64^ epiphora may cause the tear film layer to become asymmetric (thickest at the inferior margin of the pupil), leading to coma-like aberrations and decreased optical quality (vertical “comet tails") after blinking.^65^

From about 1990 until quite recently, the most common chemotherapeutic regimen for early-stage BC consisted of a 2-drug combination (an anthracycline plus cyclo-phosphamide) administered intravenously four times over a period of ∼2 months.^66^ Because the anthracycline used most often is doxorubicin (Adriamycin®), this treatment usually is referred to as"AC” chemotherapy. Anthracyclines (also including epirubicin) and cyclo-phosphamide (Cytoxan®) each interfere with DNA replication via multiple mechanisms. A prominent effect of the topoisomerase-poison doxorubicin is to intercalate DNA,^67^ while the alkylating-agent cyclophosphamide is a prodrug that after hepatic conversion leads to cross-linkages between DNA strands.^68^,^69^ The package insert for doxorubicin states that “conjunctivitis, keratitis, and lacrimation occur rarely", and while dry eye apparently due to treatment with cyclophosphamide has been reported for some non-BC patients,^70^ the package insert makes no mention of ocular or visual effects. Although at least several secondary sources cite articles reporting doxorubicin to cause watery eyes or conjunctivitis in 25% of users, the strongest statement we could locate in these earlier articles was by Blum,^71^ who commented “… some patients report increased lacrimation…”

The standard of care for early-stage BC is changing, in that taxanes now often are included in the chemotherapy regimen.^72^ Taxanes act against BC by stabilizing microrubules, thereby inhibiting mitosis.^73^,^74^ Two different taxanes- docetaxel (Taxotere®) and paclitaxel (Taxol®)- have been FDA-approved as treatments for early-stage BC, and a 4-cycle Taxotere / Cytoxan ["TC"] regimen with docetaxel has begun to replace the 4-cycle AC regimen. This change follows the 2009 publication of results from a clinical trial directly comparing the two regimens.^75^ In practice, paciltaxel tends to be used in sequential regimens, e.g., with AC administered first.^76^, ^77^ Docetaxel may be administered on either a weekly or, more commonly, a tri-weekly (i.e., once per three weeks) schedule,^78^ but the tri-weekly schedule with co-administration of cyclophosphamide is the most common regimen for early-stage BC. Docetaxel may also be given in various other regimens.^79^–^81^

There is abundant evidence showing that docetaxel often leads to epiphora.^64^,^82^ In contrast, there is limited evidence showing that paclitaxel leads to epiphora,^83^ and this limited evidence is disputed as being artifactual.^84^ Docetaxel can be present in tears,^85^ and it leads to epiphora mainly by causing canalicular stenosis in the lacrimal drainage apparatus.^86^ The severity and frequency of epiphora is less with the tri-weekly dosing schedule,^87^ but since published studies used the longer treatment regimens for metastaric BC,^87^–^89^ reported frequencies of epiphora (as high as ∼40% for tri-weekly treatments vs. ∼65% for weekly treatments)^87^ would overestimate the corresponding frequencies for early-stage (or shorter)^89^ treatment regimens. Based on our clinical experience (author SWL), about 1-2% of early-stage BC patients spontaneously report experiencing epiphora by the end of a 4-cycle TC regimen. We expect the percentage would be higher if patients were queried.

The taxanes, perhaps more so paclitaxel,^90^, ^91^ are notorious for causing peripheral sensory neuropathies,^92^ which arise at least partly from the ability of taxanes to stabilize microrubules in neurons^93^ not sufficiently protected by the blood / brain barrier.^94^ If this barrier is compromised, taxanes might affect visual function directly. Paclitaxel has been reported to alter elecrroretinographic and pattern-visual-evoked potentials,^95^ and possibly optic nerve response.^96^ There are several case reports of taxane-associated cystoid macular edema occurring in the absence of visible angiographic leakage.^97^–^102^

The first standard chemotherapeutic regimen for early-stage BC, still in use today, consists of a “CMF” drug triad of cyclophosphamide, methotrexate, and 5-fluorouracil [5-FU].^103^ The anti-metabolites methotrexate (an anti-folate)^104^ and 5-FU^105^ each interfere with DNA replication and RNA synthesis via multiple mechanisms, with each drug acting independently to inhibit thymidine synthesis. Although the anti-cancer actions of docetaxel and 5-FU are distinct, 5-FU can cause canalicular stenosis at least occasionally,^106^–^110^ suggesting an inherent vulnerability of the ocular drainage apparatus. Epiphora occurs in ∼25% or more of patients administered 5-FU,^110^–^112^ and several studies have reported systemically administered 5-FU to be present in tears.^111^,^113^ There are anecdotal reports of low doses of methotrexate leading to ocular surface inflammation,^114^ and based on clinical trial results, conjunctivitis occurs for a higher percentage of BC survivors on a CMF regimen than on an FAC regimen^115^ (i.e., conjunctivitis occurs more often when methotrexate is used in place of doxorubicin).

About 15-25% of early-stage BC patients have tumor cells that test positive for the overexpression of Human Epidermal growth factor Receptor 2 (HER2),^116^–^118^ a cell membrane protein that modulates many signaling pathways important for cell growth and proliferation.^119^,^120^ Trastuzamab (Herceptin®), a HER2-binding monoclonal antibody with multiple mechanisms of anti-cancer action,^121^ was FDA-approved in 2006 for early-stage BC, and it is now a mainstay of treatment for early-stage BC patients whose tumors test positive for HER2.^122^ Trasruzamab may be prescribed for early-stage BC in any of several chemotherapeutic combinations, which may involve periodic administration of trastuzamab for up to a year.^123^ In addition to the drugs mentioned in the preceding paragraphs, trastuzamab-containing chemotherapeutic regimens may include the platinum-based DNA adduct carboplatin.^124^ Trastuzamab apparently can cause conjunctivitis in a small percentage of patients.^125^ We can find no evidence for carboplatin affecting the eye or vision at the doses used for early-stage BC.

We conclude this section by noting: (1) that a large but somewhat controversial literature is emerging regarding the ability of cytotoxic (and endocrine) chemotherapies to induce cognitive changes collectively termed *chemobrain* (or *chemofog*),^126^–^130^ (2) that all cognitive testing batteries rely on sensory (visual and / or auditory) input to subjects or patients, and (3) that testing batteries typically include tests involving higher-order visual information processing or retrieval capabilities.^131^, ^132^ Details are outside the scope of this review.

## ADJUVANT ENDOCRINE THERAPY

As mentioned earlier, two classes of drugs- SERMs and AIs- are widely used as adjuvant endocrine therapy for women with hormone-receptor-positive early-stage BC.^8^, ^9^ The classical view is that by competitively occupying ERs, SERMs act as (a) ER-agonists or (b) ER-antagonists according to whether the SERM stimulates the ER or instead does not stimulate the ER; in the latter case the ER is prevented from functioning appropriately as it would if stimulated by an estrogen.^133^ The SERMs used against BC act as ER-antagonists in breast tissue,^134^,^135^ at least for the first several years of treatment,^136^, ^137^ and the mainstay SERM tamoxifen reduces the risk of BC recurrence for women of all ages,^138^ and also for men.^139^ The ability of SERMs to function as ER-antagonists in some tissues but as ER-agonists in other tissues depends upon many factors, including the tissue-dependent distribution of the ERα and ERβ subtypes,^137^,^140^ and even on the activity of HER2.^141^ ERα and ERβ each are present within the neural retina of men and women, with ERα apparently distributed more uniformly but exhibiting greater interpersonal variation.^20^ ERα and ERα are present also within the pigment epithelium of men and women,^19^ where tamoxifen has been reported to decrease glutamate uptake, for example.^142^ Less is known about the presence of ERs within portions of the visual system beyond the eye, and effects of SERMs on ERα vs. ERβ activity are yet to be adequately delineated for any level of the visual system.

AIs act entirely differently. By interfering with the actions of the enzyme aromatase, which catalyzes the conversions of androgens to estrogens,^6^,^28^ AIs almost completely abolish estrogen synthesis at its sources for women who are post-menopausal, whether naturally or surgically.^143^ By themselves, AIs are not effective for use in pre-menopausal women because the initial reduction in ovarian estrogen synthesis triggers feedback loops in the hypothalamic-pituitary-gonadal axis that serve to relieve this reduction.^144^ However, several clinical trials are underway to determine whether using an AI concomitantly with a gonadotrophin-releasing hormone (GnRH) agonist to disrupt this feedback system might reduce the risk of recurrence for pre-menopausal women with early-stage BC.^145^ Although male BC patients sometimes are prescribed AIs off-label, circulating estrogen levels may be reduced only incompletely for men with testicular function.^146^ Overall, the information regarding the use of AIs for male BC is quite limited.^147^–^149^

ERs may function genomically or non-genomically according to whether the ERs are present within a cell or on the cell membrane, and they may act over long (e.g., hours to days) or short (e.g., msec to sec) times-cales, respectively.^27^, ^150^–^152^ Thus, ER-dependent effects of SERMS and of AIs on vision may be mediated by either genomic or non-genomic means. However, none of the effects described in the subsequent text can be assigned confidently to one means or the other, and of course, some effects may occur indirectly, e.g. via alterations in blood flow.^153^ Moreover, several of the ocular effects in particular probably involve collateral or separate drug actions; these are identified in the text.

Before proceeding, we note that the next two sections (on the SERM tamoxifen, and on AIs) each contain some previously unpublished data from human subjects. All these data were obtained using protocols approved by the OHSU Institutional Review Board and the OHSU Cancer Institute, and all protocols were conducted in accordance with the tenets of the Declaration of Helsinki.

### The SERM tamoxifen

Three different SERMs- tamoxifen, raloxifene (Evista®), and toremifene (Fareston®)- are used to help prevent or treat BC.^135^ However, tamoxifen is the only SERM FDA-approved as adjuvant therapy for early-stage BC, and it is the only SERM approved for *every* BC stage, from the prophylactic through the metastaric settings. A fourth SERM, clomiphene (Clomid®) has long served as the standard-of-care fertility drug for inducing ovulation,^154^ and tamoxifen was used for this purpose before receiving approval as a BC treatment.^155^ Clomiphene sometimes leads to perceptible vision changes,^156^,^157^ especially when the dose is increased by a factor of up to four from the customary 50 mg / day starting dose, to achieve pregnancy.^158^ Known visual disturbances with clomiphene can include palinopsia (a prolonged afterimage, often with a trailer),^156^,^157^ photopsia (entoptic flashing lights),^158^ and scotomas.^159^ In addition, temporal resolution may be reduced slightly.^156^ Tamoxifen, clomiphene, and toremifene each have triphenylethylene structures, while raloxifene has a benzothiophene structure.^135^ The biochemical similarity of tamoxifen with clomiphene coupled with the exceedingly long ER-binding times for clomiphene^160^ raise the possibility that some of the striking effects of clomiphene may be related to visual or ocular effects of tamoxifen that are more subtle or infrequent.

Tamoxifen is mainly a prodrug in the sense that two of its many metabolites, notably 4-hydroxy-N-desmethyl-tamoxifen (endoxifen) and also 4-hydroxytamoxifen (4-OHT), now are known to have much greater affinity for ERs than does tamoxifen itself.^161^,^162^ The serum concentration of 4-OHT normally is even lower than that of endoxifen, so endoxifen is considered the most important metabolite.^161^ To the best of our knowledge, only one study has related visual-system side effects to assessment of any tamoxfien metabolites. Gallicchio et al.^163^ found that 13 of 97 tamoxifen users self-reported unspecified vision problems and that these 13 women had significantly higher serum concentrations of tamoxifen and N-desmethyl-tamoxifen (N-DMT) than did the 84 women not reporting vision problems. N-DMT is a precursor metabolite that is hydroxylated to endoxifen via action of the enzyme CYP2D6.^161^ Gallicchio et al.^163^ did not measure endoxifen levels. CYP2D6 activity varies greatly among individuals depending upon which CYP2D6 alleles a person has,^164^ and this factor with other genetic information are now considered in the development of “personalized” treatment regimens.^54^, ^55^, ^164^ HER2 status and hormone-receptor status also are factors.

(CYP2D6 is a member of the cytochrome P450 superfamily of heme-containing membrane proteins responsible for the hepatic metabolism of the large majority of clinically active drugs.^165^ See also the Redressing Side Effects section for information on CYP2D6 specifically.)

When tamoxifen was first prescribed in the late 1970s as a treatment for advanced BC, the doses were many times higher than they are now, and several case studies were published in the early 1980s concerning tamoxifen retinopathy.^166^, ^167^ Tamoxifen retinopathy classically is characterized by the presence of small crystalline deposits that may occur in the nerve fiber and inner plexiform layers near the fovea, sometimes accompanied by edema.^168^,^169^ Although tamoxifen retinopathy typically is considered to depend on total cumulative dose,^168^ spectral-domain optical coherence tomography (OCT) can reveal foveal cystoid spaces within only a year or two of the start of tamoxifen use for some patients on contemporary dosing levels,^170^ which are 20 mg / day.

The reported prevalence rates of tamoxifen retinopathy for BC survivors using standard doses vary substantially between studies, from less than 1%^171^,^172^ to about 6%,^173^ with estimates from additional studies falling within this range.^174^–^176^ This variation may be in part due to the uncertainty and / or subjectivity in detecting tamoxifen retinopathy photographically,^169^ particularly since only a few isolated crystals typically are found, and the presence of age-related changes at the fundus can make interpretation difficult.^168^, ^175^, ^176^ It is also possible that some yet undetermined factors cause incidence rates to vary across diverse populations. No subjects with tamoxifen retinopathy were identified in the 98 sets of fundus photographs evaluated for author AE's studies (2 eyes per subject, 74 of the subjects were at least 6 months amenorrheic).^177^ The subjects in Eisner's studies^178^–^184^ were selected for excellent visual acuity and normal reading vision, however, which could have contributed to the observed zero prevalence rate.

In our view, the initial findings of tamoxifen retinopathy at an auspicious time in the evolution of BC treatment have led to an overemphasis on this condition in the sense that vision symptoms (e.g., photopsia) due to other intraocular conditions may be too readily misatrributed to tamoxifen retinopathy and / or downplayed. Small numbers of crystals seem not to cause acuity loss, while mild acuity loss accompanying edema can be reversed by withdrawing the tamoxifen.^168^,^173^ The development of foveal cystoid spaces might be more serious since it may contribute to the subsequent development of macular holes,^185^ which are reported to occur in tamoxifen users at a rate about 5 times that of women not using tamoxifen.^186^ (Most of the tamoxifen users in this study^186^ were on 20mg / day but a few were on 40mg / day; Robert Bourke, MD, personal communication). Other possible causes of this elevated prevalence rate are discussed in the portion of the next section regarding virreo-retinal traction.^181^

Numerous researchers have suggested that tamoxifen retinopathy is not caused by the actions of tamoxifen on ERs, but stems instead from tamoxifen's carionic amphiphilic properties, which resemble those of chloroquine.^187^ If this suggestion is correct, then post-menopausal women who develop tamoxifen retinopathy may simply be switched to an AI for relief. Similarly, because tamoxifen, chloroquine, and other related drugs all can lead to vortex keratopathies,^188^ post-menopausal tamoxifen users who develop corneal epithelial deposits may likewise be switched to an AI. Corneal deposits have been reported in as few as 0%^169^,^175^,^189^ to as high as 72% of women using a standard dose of tamoxifen, with the presence of these deposits depending on the duration of use.^174^,^190^ The wide discrepancy arguably results from a combination of two factors: (1) differences in the sensitivity of the means by which the cornea is assessed, and (2) the subtlety of the deposits.^190^ However, other hypothetically pertinent factors (e.g., amount of sunlight exposure)^191^ might also affect the observed frequencies. Regardless, even readily observable corneal deposits are reversible^174^ and not sight-threatening.^168^

Tamoxifen can induce cataracts, particularly posterior subcapsular cataracts, in many users.^169^ In separate clinical trials regarding BC prophylaxis, tamoxifen users were found to have a significantly higher incidence of cataracts and cataract surgeries compared to a placebo group^192^ and to raloxifene users,^193^ and an independent survey of BC survivors found that women who used tamoxifen for the standard 5-year term were more likely than non-users to have seen a physician about cataracts.^194^ With one exception,^195^ studies not finding statistical significance display corresponding trends in the data nevertheless.^196^,^197^ Moreover, several animal model studies have reported that tamoxifen induces cataracts.^198^–^200^ The mechanism of cataract induction involves the blockage of swelling-activated chloride channels in the lens,^201^, ^202^ and it is at least partly independent of the actions of tamoxifen on ERs.^203^,^204^ The increased risk of posterior subcapsular cataracts may be as high a factor of 4,^169^ which is important because posterior subcapsular cataracts tend to impair function more than other types of cataracts.^205^ This visual impairment may adversely affect cognitive test performance, even at a time when visual acuity is affected minimally.^206^

Clinically evident optic neuritis resulting from tamoxifen use has been reported in only isolated cases,^174^,^207^–^210^ but the optic nerve may be affected often at a subclinical level. Eisner et al.,^178^ using scanning laser ophthalmoscopy, found that the optic cup dimensions of short-term (≤ 2 years) middle-aged (51-69 years) early-stage BC tamoxifen users were appreciably smaller than those of (a) female control subjects without BC histories and not using hormonal medications, and also (b) a corresponding group of early-stage BC anastrozole users. The cup dimensions of longer-term tamoxifen users^211^ and of younger tamoxifen users^178^ were indistinguishable from those of control subjects. (One may speculate that this latter result occurred because the younger tamoxifen users were not yet menopausal despite being amenorrheic.^212^, ^213^) The dependence of cup size on the duration of tamoxifen use is noteworthy since it is the opposite of what would occur if the effect of tamoxifen on the optic nerve head (ONH) were due to a cumulative dose toxicity. The smaller cup sizes-which were within the range of normal and thus would be regarded as unremarkable if observed on an individual basis without baseline data for comparison- were consistent with astrocytic swelling.^178^

The first study to assess the visual function of a population of tamoxifen users using measures other than visual acuity was that of Gorin et al. in 1998.^169^ They found that tamoxifen users’ color discrimination tended to be slightly compromised as assessed with the Farnsworth Desaturated Panel D-15 test. No axis of loss (e.g., tritanopic) was specified. Ritter et al.^214^ used this same test to reveal color discrimination deficits in five of six visually symptomatic tamoxifen users, and Salomao et al.^215^ reported that two of 19 visually asymptomatic tamoxifen users had diffuse color vision defects as assessed with the FM-100 hue test. Gorin et al.^169^ also reported that neither visual-field macular sensitivities (pattern 10-2) nor high- nor low-contrast visual acuities appeared to be altered. In unpublished results, we (author AE) found the contrast sensitivities of tamoxifen users (assessed with the Pelli-Robson chart) to be indistinguishable from those of female control subjects. All the tamoxifen users whom we tested met a set of strict eligibility criteria that included excellent acuity and passing a conventional color vision screening (a standard D-15 test), and no subjects showed signs of tamoxifen rerinopathy.

Even with our strict eye-health eligibility criteria in place, however, visual responses mediated via short-wavelength-sensitive (SWS) cones were found to be affected often, both in the visual-field periphery^180^ and at the fovea.^184^ In particular, visual thresholds assessed with Short Wavelength Automated Perimetry (SWAP) were related systematically to the duration of tamoxifen use, with the peripheral SWAP sensitivities of long-term users (> 2 years) lower than those of short-term users (≥ 4 months, ≤ 2 years).^180^ There was no suggestion of any corresponding effect for Frequency Doubling Perimetry (FDP),^180^ implying that a duration-of-use effect does not occur for all types of visual-field tests and suggesting that it may occur preferentially or selectively for vision mediated via SWS cones. The strong dependence of SWAP sensitivities on retinal eccentricity coupled with the strict eye-health eligibility criteria and normal contrast sensitivities make it unlikely that the observed effects on SWAP resulted from lenticular change.

Although the magnitude of the SWAP visual-field effect decreased towards the center of the visual field,^180^ SWS-cone-mediated response evidently was affected at the fovea also.^184^ That is, tamoxifen users as a group were more likely than non-BC control subjects to perceive a short-wavelength incremental test stimulus on a yellow adapting background as “white” rather than as colored. Moreover, long-term users were significantly more likely than short-term users to perceive this stimulus as “white” despite not having a selective reduction of SWS-cone-mediated sensitivity at the fovea,^184^ The absence of a foveal sensitivity loss implied that the perception of white did not result from an acquired tritanopic defect, as might otherwise have been assumed, and estimates of lens density obtained psychophysically^183^ were unrelated to the choice of color name (unpublished results). Instead, the perception of white more likely resulted from an acquired temporal-response sluggishness that caused the color from the short-wavelength test stimulus to combine, rather than to contrast, with the color from the yellow background stimulus.^182^ Given the stimulus parameters used for SWAP, a prolongation of the temporal integration (i.e., neural summation) periods for SWS-cone-mediated response would be expected to account for at least part of the long- vs. short-term differences observed in the periphery of the SWAP visual field.^179^ Related results from various human subject studies involving gender^216^, ^217^ or hormonal change^218^–^222^ collectively suggest that the ability of tamoxifen to alter SWS-cone-mediated responses involves the actions of tamoxifen on ERs.

**FIGURE 1:**
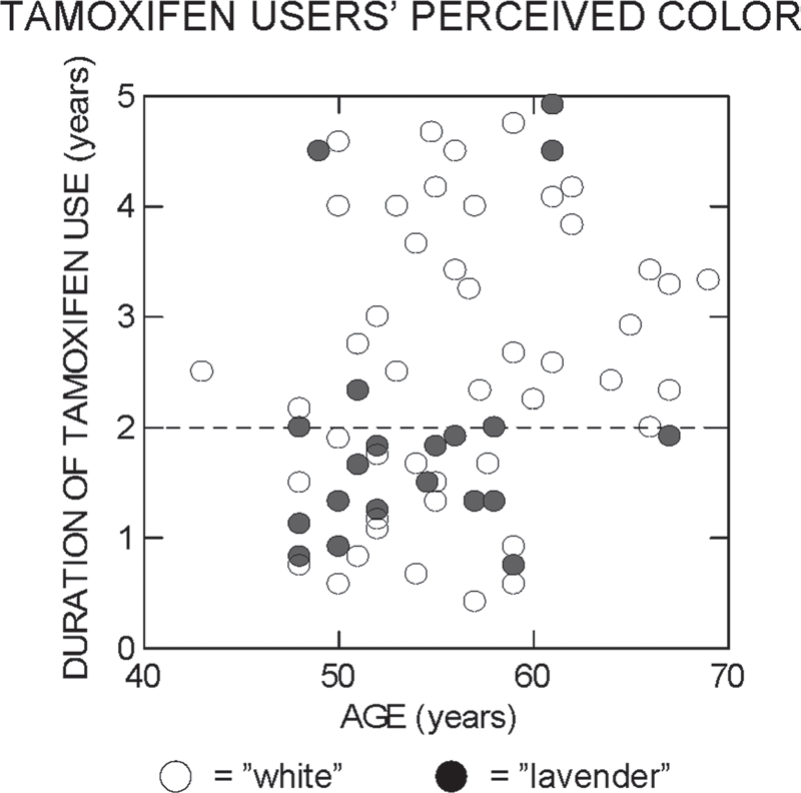
Duration of tamoxifen use versus age for the amenorrheic tamoxifen users tested with the forced-response color-naming paradigm detailed in Eisner & Incognito (2006).^184^ Open symbols represent subjects who called the threshold-level incremental test stimulus “white", and filled symbols represent subjects who called this stimulus “lavender". The test stimulus was a 3° diameter short-wavelength (440 nm) disc that was square-wave modulated at a rate of 1.5 Hz and was centered within an adapting background stimulus. The background was a moderately bright (3.6 log troland) 11° diameter, yellow (580 nm) disc viewed for at least 5 minutes. The horizontal dashed line signifies 2 years of tamoxifen use. (Data from three tamoxifen users who called the stimulus “blue” are omitted. For one of these subjects, the sensitivity was grossly reduced;^184^ the other two subjects were ages 56.8 and 59.5 years, and used tamoxifen for 4.0 and 4.9 years, respectively.) This graph has not been published previously.

The striking change in color perception at about 2 years duration-of-use (see Figure 1) in this cross-sectional study^184^ presumably resulted from changes in the body's response to sustained tamoxifen exposure, perhaps as an acquired resistance began to develop.^223^ In any event, the data provided evidence for the ability of tamoxifen to materially affect at least one CNS neural substrate, possibly by altering its timing in response to lights flashed for several hundred msec. This may have pertinence for studies of cognitive function in BC survivors using tamoxifen or other adjuvant endocrine therapies,^224^ especially if the color perception change is proven to be a consequence of reduced neural processing speed. While there appear to be no studies of clomiphene and SWS-cone-mediated response corresponding to those described in the preceding two paragraphs, the ability of clomiphene to cause palinopsia may be regarded as an extreme example of a prolonged visual response.

Little is known regarding the specific physiologic means by which tamoxifen affects SWS-cone-mediated vision or any other visual response. ERs are present in all layers of the neural retina,^20^ but how these ERs are affected individually or concertedly by tamoxifen remains undetermined. Nor is it known what roles ERs may have for supporting vision other than to contribute to neuroprotection.^225^–^227^ Interpreting the effects of tamoxifen on SWS-cone-mediated response is further complicated by the potential for cortical effects and also by the possible salience of effects not depending exclusively on the actions of ERs. The electrorerinograms (ERGs) of clinically asymptomatic tamoxifen users are affected little or not at all,^215^,^228^ but because conventional full-field and mulrifocal white-light ERGs each are insensitive to alterations of SWS-cone mediated response, functional alterations to retinal SWS cone pathways would not be evident if they existed. To understand how ER activity affects vision, it probably is more straightforward to investigate the effects of AIs rather than SERMs.

### Aromatase Inhibitors: Anastrozole (Arimidex®), Letrozole (Femara®), and Exemestane (Aromasin®)

Three third-generation AIs have received FDA-approval for use as adjuvant endocrine therapy in early-stage BC. Anastrozole (1mg / day) and letrozole (2.5mg / day) were approved in 2002 and 2005, respectively, as first-line monotherapy, while exemestane (25mg / day) currently is approved for use after 2-3 years of tamoxifen use, for a total of 5 continuous years of adjuvant endocrine therapy. In practice, however, oncologists exercise substantial discretion in deciding which adjuvant endocrine medications to prescribe to individual patients. Clinical trials are well underway to determine whether switching regimens, with tamoxifen used for the first 2-3 years and then an AI for the balance of the conventional 5-year adjuvant endocrine period, are more effective than a comparable period of monotherapy.^229^ Trials also are well on their way regarding the efficacy and safety of extending the duration of adjuvant endocrine therapy beyond 5 years.^31^,^230^,^231^

There are two broad classes of AIs: non-steroidal and steroidal. Anastrozole and letrozole each are non-steroidal AIs that by reversibly binding to the aromatase enzyme interfere with steroidal hydroxylation, thereby inhibiting estrogen synthesis.^232^ Exemestane is a steroidal AI that structurally resembles the androgen androstenedione and interacts with the substrate binding site of the aromatase enzyme, ultimately leading to the enzyme's irreversible inactivation (meaning that the subsequent production of estrogen requires aromatase to be synthesized anew).^232^, ^233^ Each AI reduces aromatization by more than 97%, with letrozole being slightly more effective in this regard than anastrozole.^230^ The effects of exemestane at this high level of suppression are more difficult to compare, for technical reasons involving the steroidal properties of exemestane.^230^

Many, but not all, of the extensively documented side effects of AIs resemble those of tamoxifen,^5^, ^31^ but relatively little is known about the effects of AIs on the visual system. The resemblance is expected to be imperfect because tamoxifen (despite often being referred to as an “anti-estrogen") is a SERM, whereas the effects of AIs on human physiology are more strictly anti-estrogenic. This difference makes AIs more useful for modeling effects of female aging on vision and the eye. One must nevertheless exercise caution when interpreting the observed effects of an individual AI such as anastrozole as an AI class effect or as an estrogen-deprivation effect. Corroborating evidence is needed, since second-order effects on testosterone levels may differ between AIs, for example.^234^,^235^

Two studies by Eisner et al. (2008)^177^ and (2009)^181^ researched the ability of anastrozole to induce retinal changes detectable ophthalmologically (A third study, Eisner et al. (2007),^178^ found that anastrozole did *not* affect the ONH, whereas tamoxifen did; this study was described in the preceding section.) These three studies all employed a cross-sectional methodology to evaluate data from early-stage BC survivors using anastrozole monotherapy. The test subjects all had excellent visual acuity, no history of eye disease or diabetes, and no high myopia; and they were younger than 70 years. Control subjects were age-matched amenorrheic women without BC histories and not using any hormonal medication. Amenorrheic BC survivors using tamoxifen mono-therapy were also tested

For the first of the two retinal studies, four of 35 (11.4%) of the anastrozole users were found to have a small retinal hemorrhage in the posterior pole of one eye, based on masked assessment of fundus photographs by an ophthalmologist.^177^ This 11.4% proportion was significantly greater than the corresponding proportions for control subjects and for tamoxifen users.^177^ Two of the four anastrozole users had a flame hemorrhage (in the nerve fiber layer), two had a blot hemorrhage (deeper in the retina), and all four apparently were asymptomatic. However, a small hemorrhage nearer to the fovea could be symptomatic. Indeed, a 47-year-old anastrozole patient who noticed a grey spot while reading was referred to an ophthalmologist who detected a small juxtafoveal hemorrhage (AE, unpublished case report). This relatively young patient, who was not a subject in our studies, was also taking a GnRH agonist.

Because small hemorrhages can resorb within months,^236^ the incidence rate of anastrozole-induced retinal hemorrhages over a 5-year period is expected to be higher than the corresponding prevalence rate based on a cross-sectional snapshot, which provided the basis for the 11.4% rate reported by Eisner et al. (2008)^177^ for their small but select sample. On the other hand, the hemorrhages observed by Eisner et al. (2008)^177^ might have not have resulted solely or directly from the AI itself. Instead, they might have arisen at least partly from the actions of additional drugs used to relieve muscle or joint pain (aspirin) or to preserve bone density (bisphosphonates).^177^ This possibility is discussed in greater detail and in a more general context in the next section. Because the retinal hemorrhages were subtle, comparable hemorrhages might be missed in a cursory eye exam, or in an eye exam by someone other than an eye care specialist. The hemorrhages observed by Eisner et al. (2008) did not result from high blood pressure,^177^ but excessive traction pulling on the retina may have been a factor,^181^ as menopause appears to increase the risk for several vitreo-retinal fractional conditions.^237^–^241^

**FIGURE 2:**
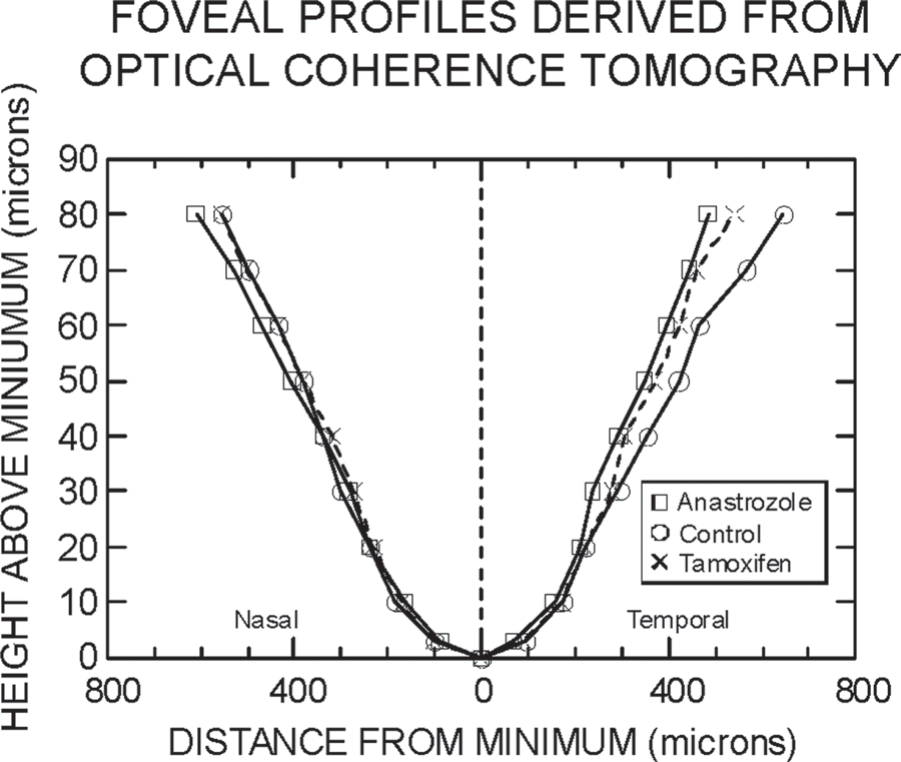
Foveal shape profiles normalized to the locus of minimal retinal thickness; nasal direction is to the left, and temporal to the right. Squares (□) represent median data of anastrozole users, circles (O) represent median data of control subjects, and crosses (x) represent median data of tamoxifen users. Connecting lines are unbroken for the anastrozole users and control subjects, and dashed lines are for the tamoxifen users. All units are microns (μn). Note that the scales on the two axes differ. The locus of minimal thickness defines a height of 0 μm on the ordinate. Data are derived from subjects without detectable PVDs. This figure was previously published as Figure 1 in (Eisner, Thielman, Falardeau, Vetto Vitreo-retinal traction and anastrozole use. *Breast Cancer Res Treat.* 2009;117:9-16),^181^ but with a different legend and title. The graph is reproduced with kind permission from Springer Science+Business Media B.V.

To observe and quantify effects of traction on retinal anatomy, Eisner et al. (2009)^181^ transformed retinal thickness data obtained using time-domain OCT into foveal shape profiles corresponding to a slice along the horizontal (i.e., nasal / temporal) meridian. The effects of traction between the vitreous and retina then could be measured and compared across subject groups. As predicted, the foveal shapes of anastrozole users were distorted in a manner consistent with a heightened degree of vitreo-retinal traction, with the upper portion of the temporal side of the foveal slope displaced towards the ONH, an asymmetrically located major anchor for the vitreous.^242^ The results^181^ are shown graphically in Figure 2. The nasal-temporal asymmetries differed significantly between anastrozole users and control subjects, but the corresponding asymmetries for tamoxifen users appeared to be intermediate to those of the other two groups and the statistical comparisons were inconclusive.^181^

The data for Figure 2 were analyzed for subjects without discernible posterior vitreous detachments (PVDs), and in fact, the tamoxifen users without PVDs were observed to have used tamoxifen for a significantly shorter duration than the tamoxifen users with PVDs.^181^ (Most subjects’ PVDs evidently were partial rather than complete.^181^) Among anastrozole users, it is likely that the heightened degree of traction resulted from estrogen depletion, since PVDs^237^ and macular holes^238^, ^239^ each are traction-related conditions that occur more often in women than in men, and moreover, may be precipitated by the menopausal transition.^240^, ^241^ Estrogen supplementation might even protect against the development of macular holes.^238^ Thus, AIs may increase the risk of macular holes or other relatively acute traction-related conditions such as rhegmatogenous retinal detachments.^243^ Any patient reporting worsening floaters or photopsia should be promptly referred by their oncologist to an ophthalmologist. In our clinical experience (author SWL), about 1 in 30 AI users mention that these entoptic phenomena develop or worsen with AI use.

Eisner et al.^181^ noted in retrospect that their anastrozole subjects were, on average, less myopic than were their subjects in the tamoxifen and control groups. This observation suggested a possible recruitment bias wherein disproportionately many myopic anastrozole users did not enroll in the study, possibly because they felt their vision was no longer normal. Epstein^244^ subsequently published a case report on the deterioration of visual acuity in two myopic BC survivors who switched from a non-steroidal AI to exemestane; one developed a macular hole after the switch. Myopia is a known risk factor for PVDs^237^ and macular holes,^245^ and when combined with excessive vitreo-retinal traction, a shearing force can be created at the fovea.^181^ In 2004, the Intergroup Exemestane Study (IES) had reported that early-stage BC survivors who had switched from tamoxifen to exemestane after 2-3 years were significantly more likely to develop visual disturbances than were women who remained on tamoxifen, but the types of disturbances were not specified.^246^ It would be desirable and feasible to test BC survivors before

and after they switch from tamoxifen to an AI in order to determine whether the switch leads to measurable intraocular change, particularly as assessed with spectral-domain OCT.

The eye is unique among all the organs of the body in that its interior can be viewed and measured with an extraordinarily high degree of precision. Thus, changes in intraocular anatomy are potentially useful for marking and tracking changes elsewhere in the body that result from AI use. Of all AI side effects, the one most often leading to medication non-compliance is arthralgia (joint pain).^247^ The strong degree of biochemical similarity between joints and vitreous^242^,^248^–^250^ raises the possibility that measured changes in vitreo-retinal traction might be useful in helping to mark changes in joints that accompany the development of AI-induced arthralgia,^251^, ^252^ and that are likely to result from a decrease in estrogen synthesis.^253^ This is a matter for future study.

The same types of color perception changes experienced by tamoxifen users^184^ also were experienced by anastrozole users,^182^ but with an important difference. That is, whereas the effects of tamoxifen depended on the duration of use,^184^ the effects of anastrozole depended on age, with the anastrozole users who perceived the test stimulus as white being significantly younger (by about 6 years on average) than the anastrozole users who perceived it as colored.^182^ Because estrogen production normally is greater for younger post-menopausal women than for older post-menopausal women,^254^, ^255^ this result suggests that the anastrozole-induced change in estrogen exposure, rather than just the low estrogen levels themselves, led to the perception of white.^182^ Bear in mind that because the perception of white was elicited for a very specific set of laboratory conditions (a short-wavelength test stimulus flashed on a larger, moderately bright, yellow background), this perception need not reflect either a more generalized or real-word color vision deficit wherein hues appear desarurated. Instead, the perception of white more likely manifests a reduction of processing speed within the functionally isolated neural substrate responsible for SWS-cone-mediated vision.^182^ Future studies should directly test the hypothesis that AIs prolong the temporal integration (neural summation) periods for SWS-cone-mediated response, since if AIs cause SWS-cone-mediated response to become more sluggish, the likelihood is strong that the temporal response properties of some other CNS neural substrates are altered similarly. Cognitive processing speed, for example, may be slowed by anastrozole use,^256^, ^257^ and senses such as hearing that depend exquisitely on response timing might also be vulnerable to reductions of estrogenic activity.^258^,^259^

In unpublished results (author AE), the monocular contrast sensitivities of anastrozole users were found to be significantly lower (average difference = 0.08 log units, corresponding to ∼½ line of loss on the Pelli-Robson chart) than those of female control subjects and tamoxifen users, respectively. Thus, the spatial vision of anastrozole users was affected despite their excellent acuity. Contrast sensitivity should be assessed for AI users reporting unexplained vision difficulties.

One final note: Although chronic dry eye can appreciably impact quality of life,^260^ and there are physiological and epidemiological reasons for supposing that AI usage can lead to dry eye, only one study appears to have expressly raised this possibility.^261^ ERs are present in the cornea^16^ and tear film glands,^16^, ^18^, ^22^, ^23^ conditions such as premature ovarian failure apparently can lead to dry eye,^262^ and dry eye is most prevalent among post-menopausal women.^3^,^62^ While these considerations suggest that the abrupt reduction of estrogen synthesis can lead to dry eye, the relation of hormonal activity to dry eye evidently is more complex, as it is now seems likely that androgen / estrogen balances are more important than estrogen exposure alone for maintaining the ocular surface and for minimizing ocular surface discomfort.^3^, ^263^,^264^ Either view justifies conducting studies to determine whether AIs may contribute to the development or exacerbation of dry eye. An objective and practical measure of tear film osmolarity has been reported recently to correlate well with dry-eye severity as assessed subjectively,^265^ so we propose that studies relating tear film osmolarity to BC treatment be conducted along with administration of a validated survey tool. Osmolarity measurements can be performed quickly and easily, and hence can be appended to clinical trials or other studies.

## MEDICATIONS USED FOR REDRESSING SIDE EFFECTS OF BC TREATMENTS

Hot flashes are a relatively common side effect of tamoxifen and of all the AIs.^5^,^31^ Because supplemental estrogen is contraindicated for most BC survivors, they are instead often prescribed serotonin specific reuptake inhibitors (SSRIs) for symptomatic relief.^266^ SSRIs also are prescribed as anti-depressants.^267^ However, at least several different SSRIs, notably paroxerine (Paxil®) and fluoxeteine (Prozac®) but not citalopram (Celexa®), strongly interfere with the ability of CYP2D6 to convert tamoxifen to its active metabolites, particularly endoxifen.^162^,^268^ Consequently, women using tamoxifen are no longer routinely prescribed SSRIs that are potent CYP2D6 inhibitors. SSRI usage remains common among tamoxifen and AI users, and several percent of SSRI users overall have been reported to complain of “blurry vision,” possibly due to a mydriatic effect.^269^,^270^ Interestingly, metabolism of the intraocular pressure drug timolol depends on the activity of the same CYP2D6 enzyme as tamoxifen, so patients with low CYP2D6 activity may be at increased risk for beta-blocker induced brachycardia following systemic absorption of ophthalmically-administered rimolol.^271^,^273^ Oncologists should be aware that the glucocorticoid dexamethasone, one of several types of drugs used for anti-emesis while patients are receiving chemotherapy,^274^ can acutely raise intraocular pressure in susceptible people.^275^,^276^

Because AIs reduce bone density, women who are prescribed an AI are sometimes also prescribed a bisphosphonate,^277^ a type of medication that decreases bone resorption by disrupting osteoclast function.^278^ However, bisphosphonates can have serious side effects,^279^ so they are not prescribed merely as a matter of course.^280^ It has become clear that bisphosphonates, especially the ones administered intravenously, occasionally lead to clinically significant uveiris and scleritis, generally of the anterior portion of the eye.^281^–^283^ The intravenously administered bispshosphonate zolen-dronate (Zometa®) may have anti-cancer properties in its own right,^284^ so several clinical trials are underway to determine whether and how zolendronate should be added to existing AI regimens.^285^ The preliminary results regarding upfront use of zolendronate (administered at 6-month intervals) combined with daily AI use indicate that BC recurrence rates are reduced.^286^ Thus, the use of zolendronate is likely to increase in the future, in which case, the rates of uveitis and scleritis among BC survivors will also likely increase.

The small retinal hemorrhages detected in a significant proportion of anastrozole users^177^ (see the previous section) might also have depended in some cases on the use of supplemental medications for alleviating AI-induced side effects. Of the four out of 35 anastrozole users with retinal hemorrhages, the two with blot hemorrhages were the only anastrozole users also using both a bisphosphonate (taken orally) and aspirin.^177^ These limited data suggest that blot hemorrhages can result from a drug combination that includes an AI and a bisphophonate acting synergistically on the posterior portion of the eye. Bisphosphonates may affect the posterior uvea at a greater rate than is recognized,^287^ and estrogen depletion might increase retinal vascular permeability.^288^

## DIRECTIONS FOR FUTURE RESEARCH

Although treatments for BC are continually evolving, it seems certain that millions of BC survivors will receive adjuvant endocrine therapy involving years of AI use. What remains unknown is the scheduling of this treatment. Will AIs be prescribed after 2-3 years of tamoxifen use, or will AIs be used from the outset? Will patients receive adjuvant endocrine therapy for more than 5 years? Which AIs will be used, and how might their visual and ocular effects differ? How might prior use of tamoxifen interact with the ability of an AI to affect vision and the eye? This last question is important because many post-menopausal women may be using tamoxifen for the first several years at the start of a tamoxifen / AI switching regimen, while many other women will have taken tamoxifen for varying periods of time before reaching the menopause. Tamoxifen will continue to serve as an alternative medication for post-menopausal women whose AI side effects are unacceptable, and it is expected to remain a mainstay for men with BC.

Because iatrogenic (treatment-induced) changes to vision or the eye may impact BC survivors’ quality of life and hence reduce adherence to treatment, such changes may indirectly cause BC recurrence rates to increase. Iatrogenic changes may also sometimes be misattributed to natural aging. Thus, studies are needed to determine whether AIs contribute to the development or exacerbation of dry eye, and if they do contribute, then which AIs contribute most and in which regimens. These dry-eye studies should include measures of tear-film osmolarity. We also suggest that studies be conducted to evaluate the ability of AIs to produce traction capable of altering the existing relation between the vitreous and retina. These studies should include quantitative assessments of foveal shape indices derived from retinal thickness measurements made with spectral-domain OCT devices. Assessing virreo-retinal traction is important because excessive traction (a) may produce entopric symptoms that disturb or concern patients in the short-term, and (b) may raise the risk for subsequent sight-threatening conditions such as macular holes. Perhaps even more importantly, evidence is accumulating to suggest that increased traction may raise the long-term risk for blinding conditions such as macular degeneration.^38^, ^289^–^291^ Thus, we recommend that oncologists routinely query AI users about the development of traction-related symptoms such as floaters or photopsia. We also recommend that AI users be queried about changes in ocular surface discomfort and that contrast sensitivities be routinely assessed, which is feasible in an oncology setting. Worsening of any these factors may warrant referral to an ophthalmologist.

Because drugs that drastically reduce ER activity can be administered to people only with compelling clinical justification, the widespread use of AIs provides a unique chance to investigate the means by which estrogen deprivation (or conversely, estrogen exposure) affects the adult human visual system, particularly for women at and beyond menopause. These investigations should include studies of the etiology of traction-related intraocular conditions such as PVDs and macular holes. Moreover, because the eye is so accessible and visual response is so quantifiable, measures of the eye and vision might *also* be assessed for their ability to mark related iatrogenic changes occurring in parts of the body that are more difficult to view and / or quantify. For example, the use of OCT to quantify AI-induced anatomical changes at the vitreo-retinal interface might be investigated as a means for marking coincident changes occurring in joints^247^ (and which have long been postulated to share a common etiology with eye changes^292^, ^293^). Because commercial OCT devices are widely distributed and are not intimidating to patients, OCT is well-suited

for use in longitudinal studies, whether conservative or more speculative.

Similarly, future investigations should include assessments of visual functions susceptible to changes in estrogenic activity. Because SWS-cone-mediated visual response is important only for color vision and not, for instance, for fine spatial resolution,^294^ AI-induced changes in SWS-cone-mediated response^182^ are not expected to compromise a person's ability to function in her environment. By the same token, however, SWS-cone-mediated response sensitivities can be measured using established procedures that provide an exceptionally high degree of functional isolation,^216^, ^295^, ^296^ thereby allowing the response dynamics of an operationally well-defined neural substrate to be measured behaviorally with little or no intrusion from other neural substrates^182^ (which do not share the same pre-corrical anatomic pathways dedicated to SWS-cone-mediated vision^297^, ^298^). If the temporal integration periods of SWS-cone-mediated response are prolonged by AI use, as we hypothesize, there is only a remote chance that no other neural substrate is made more sluggish in this way. We therefore suggest that psychophysical studies be conducted to determine whether AIs cause the temporal integration periods for SWS-cone-mediated response to become prolonged. (Corresponding fMRI studies may be performed,^299^, ^300^ but because of practical constraints, fMRI studies are limited to small numbers of subjects.) If our hypothesis is supported, a next step is to determine whether response sluggishness measured for SWS-cone-mediated vision can be used to mark deficits in cognitive processing that may result from one or more adjuvant endocrine therapies.^224^ For the present, we recommend that studies of BC survivors’ cognitive function begin to record or screen for eye health and also add tests of spatial vision so that sensory deficits are not misconstrued as cognitive deficits.^206^

## Notice of correction

The Early Online version of this article published online ahead of print on 5 August 2011 contained errors.

1. The start of the Abstract on page 867 has been changed from “This review concerns the visual and ocular effects…” to “This review concerns the effects on vision and the eye…”
2. The original receipt date has been added at the bottom of page 867.
3. Capitalization has been corrected in the section heading on page 873.

These errors have been corrected for the current version.
